# Emotionally Responsive Teaching in Aging Education: A Framework for Transformative Pedagogy

**DOI:** 10.1093/geroni/igaf122.4371

**Published:** 2025-12-31

**Authors:** Juanjuan “June” He

**Affiliations:** Drexel University, Philadelphia, Pennsylvania, United States

## Abstract

As aging education increasingly intersects with design, community practice, and care, the emotional dimension of teaching remains under-recognized. This presentation introduces a framework for emotionally responsive teaching—a pedagogical approach that centers emotional presence, vulnerability, and cultural humility as catalysts for transformative learning. Grounded in a multi-year Aging & Design course series, this framework emerged from immersive collaborations between university students and older Asian immigrants in Philadelphia. Students engaged in community-based co-design not only developed inclusive design responses, but also confronted personal assumptions, ageism, and deep cultural difference. Through structured reflection and experiential learning, students showed significant shifts in empathy, cultural awareness, and their understanding of aging as a lived and social experience. The presentation shares practical strategies for cultivating emotionally attuned classroom environments, drawing from student reflections, pedagogical tools, and qualitative and quantitative insights. It proposes that emotionally responsive teaching is not ancillary, but foundational: enabling students to engage more fully, listen more deeply, and reimagine aging not just as a demographic issue, but a human one. This session is designed for educators in gerontology, design, social work, and health professions seeking to bridge knowledge and care, and to reshape the classroom into a space of intergenerational connection, inclusion, and ethical growth.

